# Dermal open flow microperfusion for PK-based clinical bioequivalence studies of topical drug products

**DOI:** 10.3389/fphar.2022.1061178

**Published:** 2022-11-22

**Authors:** Thomas Birngruber, Katrin I. Tiffner, Selma I. Mautner, Frank M. Sinner

**Affiliations:** HEALTH—Institute for Biomedicine and Health Sciences, JOANNEUM RESEARCH Forschungsgesellschaft mbH, Graz, Austria

**Keywords:** bioavailability, topically applied dermal drug products, verification study, cutaneous PK, dermal penetration, PK-based BE study, interstitial fluid, systemic redistribution

## Abstract

Topically applied drug products have experienced an extraordinary price increase in the United States, mostly due to a lack of generic products. Generic drug development is hindered by high costs and risks associated with clinical endpoint studies required to show bioequivalence (BE) of prospective generic products relative to their reference products. There is a continued need for cost- and time-efficient alternatives to clinical endpoint studies to determine BE of topically applied dermal drug products. Cutaneous PK-based BE studies present such an alternative and dOFM (dermal open flow microperfusion) has already been successfully used in several verifications studies to show an accurate and sensitive assessment of the rate and extent at which drugs become available in the skin. dOFM technology is discussed as well as the dOFM setup of clinical pilot and main studies to achieve BE assessment with a minimum number of participants and an outlook is given on the use of dOFM technology for other drug products.

## Introduction

A price increase for dermal drug products of 279% in the years 2011–2014 (Rosenberg and Rosenberg, 2016) reflects the urgent need for high-quality and lower-cost generic drug products, especially in the United States. Generics offer the chance of providing efficient and affordable topical drug products but currently 80% of topical drug products have fewer than three approved generics (Ramezanli, 2019). This situation is believed to originate from the relatively small market size of topical drug products and also from the very high costs and high failure risk of comparative clinical endpoint studies that have to demonstrate bioequivalence (BE) of a generic drug product and a reference product.

Until recently, clinical endpoint studies for BE assessment were required by the United States Food and Drug Administration (US-FDA) with only very few exceptions. These exceptions are based on pharmacodynamic endpoint studies, i.e., a vasoconstrictor study (Raney et al., 2022) or on the concept of sameness/similarity of the topical formulation regarding components (Q1), composition (Q2), and arrangement of matter (Q3). A generic drug product fulfilling Q1/Q2 sameness has identical components and composition (±5%) relative to the reference drug product and has thus a minimized possible failure risk in terms of e.g., stability and solubility of the drug product, irritation and sensitization, or formulation interaction with diseased skin. A generic drug product showing Q1/Q2/Q3 sameness to its reference drug product may even qualify for an *in vitro* waiver. For example, the “Draft Guidance on Acyclovir Cream” allows an *in vitro* assessment if the generic and the reference product show Q1/Q2 sameness and if both products are physically and structurally similar. For this *in vitro* assessment both products must show equivalent acyclovir release rates (shown by *in vitro* release testing) and must also demonstrate BE based on an acceptable *in vitro* permeation test (US Food and Drug Administration, 2018). Recently, the concept of Q1/Q2/Q3 sameness has been further advanced. US-FDA introduced the concept of similarity where components, composition, physical, and structural properties of the generic drug product have to be similar but not the same relative to the reference drug product. This similarity concept allows for more variation of the generic drug product in terms of components and composition of the formulation as well as its arrangement of matter as long as there is no significant effect on local or systemic bioavailability compared to the reference drug product.

For drugs with Q1/Q2/Q3 similarity, an *in vitro* waiver may not be applicable and bioavailability can be determined by using PK-based BE studies (US Food and Drug Administration, 2013). However, for topical generic drug products that are not intended to be absorbed into the bloodstream, the standard blood-based *in vivo* PK studies might not be suitable since levels of the active pharmaceutical ingredient (API) in blood samples are often too low to be detected and API concentrations in the blood do not necessarily reflect the API concentration at the site of action. For topical generics, comparative dermal *in vivo* PK studies provide an alternative to assess BE of a generic drug product relative to its reference product. These alternative dermal PK-based studies offer the highest potential for cost savings and, by replacing expensive and time-consuming clinical endpoint studies, provide a promising option for the development of affordable generic topical drug products. Dermal PK-based methods such as dermal open flow microperfusion (dOFM) are thus innovative and promising alternatives to address BE of any dermal topical drug product, including products that do not meet Q2/Q3 sameness and complex drug products and delivery systems which cannot apply for the other approaches.

In this review we discuss dOFM as an innovative tool for dermal PK-based BE assessment in dermal generic drug approval. We present the methodological background, introduce a general clinical study design for BE assessment and show a successful case study.

## Dermal open flow microperfusion-dOFM

Open flow microperfusion (OFM) is based on a membrane-free probe design to be used for continuous sampling in different tissues (Bodenlenz et al., 2012; Pieber et al., 2012; Birngruber et al., 2013; Höfferer et al., 2015; Tiffner et al., 2017). dOFM is one specific OFM application which is optimized for dermal studies and which is CE-certified for use in clinical studies in the European Union (Bodenlenz et al., 2013). The linear dOFM probe (probe diameter 0.55 mm) features an open exchange area made from a PEEK (polyetheretherketone) mesh with macroscopic openings (0.2 mm) that enable direct contact of perfusate with the surrounding interstitial fluid (ISF).

**FIGURE 1:**
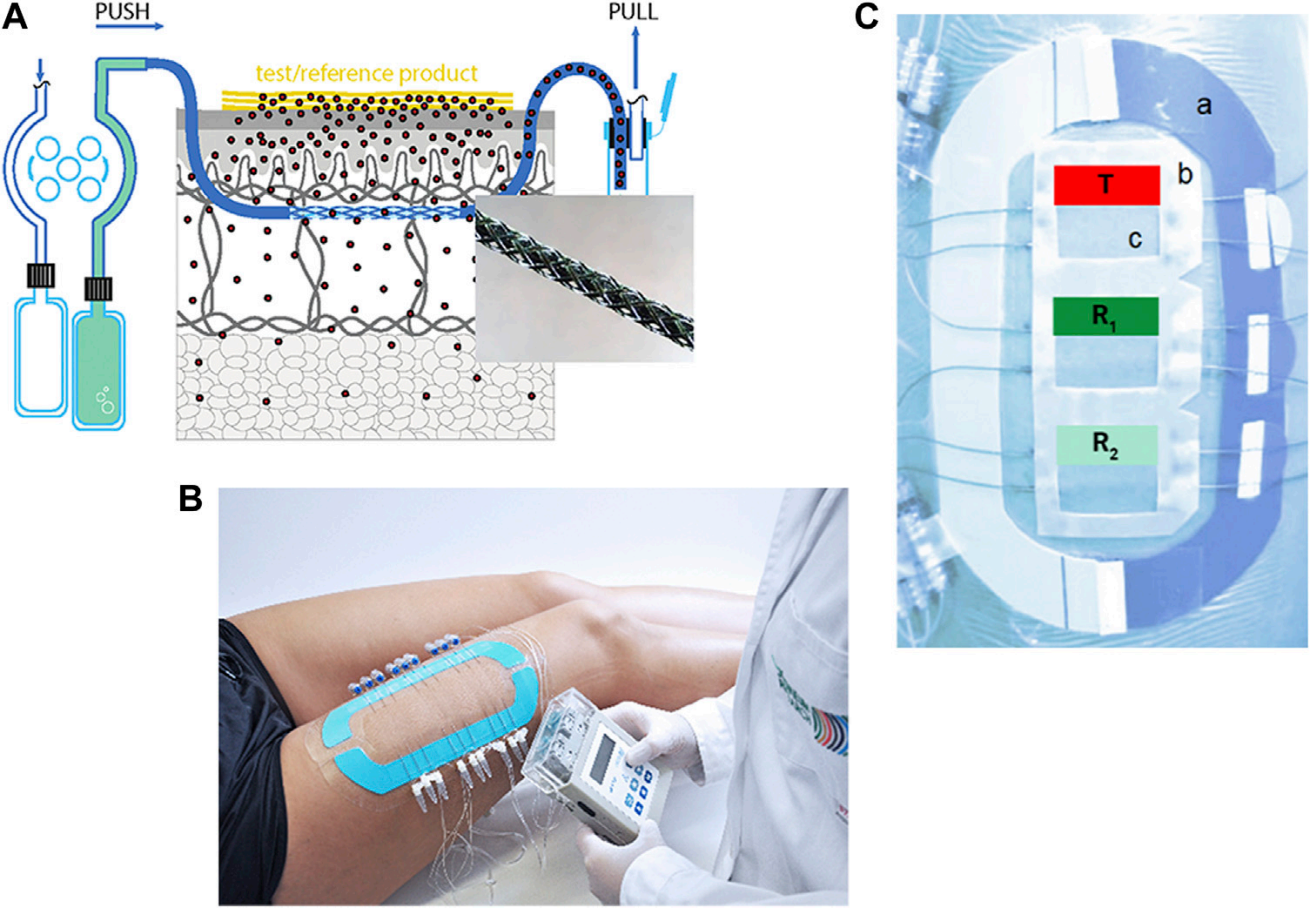
**(A)**: Schematic overview of dOFM sampling setup with enlargement of the exchange area. **(B)**: Complete clinical setup with three application sites, nine inserted dOFM probes and a wearable pump. **(C)**: A stabilization ring (a) is used to minimize skin deformation. A standardized application frame (b) exactly defines the application site (c). T (test drug product), R_1,2_ (reference drug product).

The probes are inserted into the dermis under highly standardized conditions (application sites with a defined location and size, stable temperature, minimal probe depth variation verified by ultrasound measurements) and connected to a wearable push/pull pump (Figure 1B) that allows a wide range of flow rates (0.1–10 μl/min). The probes are perfused with a physiological solution (perfusate) which equilibrates with the surrounding ISF. dOFM samples consist of diluted but unfiltered ISF and thus contain all chemical information of the dermal ISF. Due to the rather small sample volumes (e.g., a perfusion rate of 1 μl/min generates a dOFM sample with a volume of 60 μl in 1 h) dOFM samples have to be analyzed in an optimized analytical setup that includes specific sample preparation steps to remove proteins from the complex matrix of the dOFM samples without depleting the API of interest (Schimek et al., 2016; Schimek et al., 2018). Further, possible enzymatic degradation of the API of interest by certain matrix components that may bias the results must be taken into account.

## Clinical PK-based dOFM studies

All clinical dOFM BE studies assess the dermal PK parameters area under the dermal concentration curve (AUC), dermal peak concentration (C_max_) and time to reach C_max_ (t_max_) of the concentration-time profiles in dermal ISF samples similar to PK-based BE studies that use blood samples for testing orally administered drug products (US Food and Drug Administration, 2013). dOFM studies can be used to investigate dermal PK of any drug product and any formulation, e.g., creams, ointments, gels, or transdermal patches. A typical cutaneous dOFM BE assessment comprises a pilot and a main study. When little data is available about the dermal PK of the drug product, *ex vivo* studies in human skin explants (Hummer et al., 2022) and preclinical dOFM studies are performed prior to the pilot study. Any dOFM study is performed with the same dOFM setup/material irrespective of the *ex vivo*, preclinical or clinical setting.

In the clinical dOFM pilot study, parameters such as dose sensitivity, sampling duration, lateral diffusion, systemic redistribution, and analytical calibration range are verified prior to the main study. Computer modelling based on pilot study results can be used to support the design of the main study and can also help to estimate the number of participants required for sufficient statistical power in the main study.

### dOFM pilot study design

In the dOFM pilot study, the reference product (and the test product if already available) is generally tested in four to eight healthy participants. To assess dose sensitivity, the pilot study is designed to detect changes in dermal bioavailability relative to different dose amounts of the drug product. The selected dose should not surpass the bioavailability threshold, where a further dose increase would no longer increase the bioavailability in the dermis.

The dOFM pilot study is also used to verify optimal sampling duration and sampling intervals. Depending on the drug product, C_max_ can either occur instantly after application or it can show a lag time of several hours. Hence, sampling duration and sampling intervals are optimized in the dOFM pilot study to accurately detect C_max_. In order to successfully calculate AUC, the sampling duration has to be optimized to also include the decline of the dermal concentration-time profile.

Lateral diffusion of drug products between adjacent application sites can influence BE assessment (Gee et al., 2012; Nguyen et al., 2018). The dOFM pilot study monitors any lateral diffusion by placing one additional non-dosed application site next to one of the dosed application sites to assess the amount of lateral API diffusion occurring between these sites.

Some highly permeable, topically applied drug products are absorbed into the systemic circulation *via* lymphatic and capillary vessels and are then redistributed into the dermis. Systemic absorption and systemic redistribution are also measured in the pilot study to minimize the risk of drug background levels that could affect dOFM BE assessment in the main study. In the pilot study, blood samples are taken after topical application of the reference product to monitor systemic absorption. Systemic redistribution is then assessed by analyzing dOFM samples from an additional non-dosed site that is placed at a reasonable distance (e.g., on arm) from the dosed application sites on the thigh.

### dOFM main study design

In the main clinical dOFM BE study, the generic test product and the reference product are tested in parallel by applying them on adjacent application sites in the same healthy participant. This design reduces inter-subject variability and thus lowers the number of participants needed for clinical PK-based dOFM studies relative to clinical endpoint studies (Bodenlenz et al., 2020). A high level of standardization in each study is necessary irrespective of the tested drug products.

Generally, the application sites are located on both thighs. Additional application sites can be added depending on the research question, e.g., an application site on the upper arm to monitor systemic redistribution. The use of a standardized application frame (Figure 1C (b)) with cut-out application sites ensures exact placement of dOFM probes and an accurate and reproducible application of the drug products. A self-adhesive stabilization ring (Figure 1C (a)) is positioned around the application sites to avoid deformation and excessive stretching. Before dOFM probe insertion, sterile ice bags are applied to minimize any pain and avoid the use of anesthetics that may influence dermal metabolism (Bodenlenz et al., 2012; Bodenlenz et al., 2013). Tolerability of probe insertion has been tested in a previous study to ensure that a clinical setup with multiple probes is well accepted by study participants (Bodenlenz et al., 2013).

Exact dOFM probe placement in the skin is confirmed by ultrasound measurement and minimizes data variability due to probe depth variations. After probe insertion, the skin is cooled again for approx. 1 h of sampling to minimize trauma formation. After this run-in phase, the generic test product and the reference product are applied in a blinded manner in a randomized order. Application also follows a highly standardized workflow to minimize any variation due to product application. The drug products are applied in close proximity to each other to increase the probability of similar skin characteristics. Continuous dOFM sampling is started immediately after product application and is maintained for up to 48 h. dOFM samples are typically taken every 60 min. Sampling intervals can be varied depending on the PK profile of the tested drug. If required, the application sites can be occluded and/or the drug products can be removed at any specific time during sampling. Additionally, blood samples are collected to monitor potential systemic absorption.

After sampling is completed, dOFM samples are frozen for bioanalytical analyses, e.g. HPLC-MS/MS after SPE clean-up (Schimek et al., 2018). From the measured concentration-time profiles, the dermal PK parameters AUC, C_max_, and t_max_ are derived. These PK parameters are the same as those used for blood-based BE assessment of oral formulations and thus the same statistical methods are used for PK-based BE assessment (US Food and Drug Administration, 2013). The average BE (ABE) approach is the standard statistical BE testing approach for blood-based BE assessment. ABE uses AUC and C_max_ to compute individual ratios for each participant regarding the test drug product and the reference product. A 90% confidence interval (CI) is computed based on the geometric mean ratios and BE is confirmed if the CI lies within the limits of 80% and 125%. As topical drug products usually exhibit highly variable skin PK they qualify for the scaled average BE (SABE) approach (US Food and Drug Administration, 2021). SABE additionally scales the BE limits to the intra-subject variance of the reference product. The appropriate statistical approach (ABE or SABE) is selected based on the within-reference variability. SABE is used if the within-reference variability is higher than 0.294 and ABE is used if the within-reference variability is smaller or equal to 0.294. Using SABE instead of ABE for topical drug products results in a lower number of participants in the main study. Both, ABE and SABE have been successfully used to analyze dOFM data (Bodenlenz et al., 2017; Tiffner et al., 2020)

## Strengths of dOFM for PK-based BE studies

dOFM can be used to sample any API regardless of its molecular size or structure or the formulation that is used for its delivery. dOFM has already been successfully used to sample lipophilic (Bodenlenz et al., 2012; Holmgaard et al., 2012; Bodenlenz et al., 2016), high-molecular-weight (Dragatin et al., 2016; Kolbinger et al., 2017) and hydrophilic APIs with low skin permeation ability (Bodenlenz et al., 2017), bound and unbound drugs, peptides, proteins, antibodies, and APIs with high-protein-binding properties (Dragatin et al., 2016; Kolbinger et al., 2017; Hummer et al., 2020; Tiffner et al., 2020). dOFM samples reflect the molecular composition of the ISF because sampling collects diluted but unfiltered dermal ISF. The membrane-free design minimizes the risk of affecting the measured API concentration and subsequent BE results. dOFM samples also have a minimal risk of being contaminated compared with biopsy samples.

dOFM studies excel by continuous sampling for long study durations due to the wearable pumps that allow study participants a limited mobility (e.g., bathroom breaks) and due to membrane-free probes that are not affected by membrane-clogging or fouling during long sampling intervals. This continuity of dOFM sampling generates concentration-time profiles obtained from the same application site from sampling start to sampling end. dOFM is CE-certified to be used for clinical studies up to 48 h and has successfully been used to show stable sampling for 36 h (Bodenlenz et al., 2017). Long sampling is also supported by a very low inflammation risk and only minor discomfort has been reported by participants (Bodenlenz et al., 2013). Generally, in none of the studies any adverse event has been reported with regard to the inserted dOFM probes.

In addition, dOFM studies have also shown a low intra-subject variability (9%–18% log AUC) (Bodenlenz et al., 2020) indicating reproducibility due to high methodological standardization. All dOFM material and study procedures have been thoroughly optimized and highly standardized in the last few years which includes the use of template frames for probe placement, skin pre-cooling procedures, probe depths monitoring (by ultrasound), room temperature and humidity control, skin care before the study, implantation procedure, and substance application procedure, all of which led to high‐quality data that enable successful BE assessment.

One of the main strengths of PK-based dOFM BE studies is the considerably lower number of participants (twenty to thirty) compared with clinical endpoint studies (hundreds to thousands). By using the standardized dOFM setup, test and reference product can be tested (even with replicates) at the same time in the same participant. Simultaneous testing in one participant reduces inter-subject variability and is thus a major advantage over study designs that are limited by high inter-subject variability. If, in addition, systemic redistribution of the tested drug products is low, application sites on the two thighs of one participant are considered to be independent.

dOFM studies are limited to sampling in the dermis and although dOFM is much less invasive than other sampling techniques (e.g., biopsies), data interpretation has to consider a potential influence of probe insertion.

## dOFM use case for PK-based BE assessment

In a US-FDA co-funded **verification study**, dOFM was tested to determine whether it showed the necessary accuracy and reproducibility to assess BE for acyclovir creams (Bodenlenz et al., 2017). *In vitro* release testing, *ex vivo* and preclinical studies were used to obtain first data on dermal PK of acyclovir and to select a negative, non-BE control drug product that has a different bioavailability than the reference product.

In the dOFM **verification pilot study**, dOFM was used in six healthy participants to determine dose sensitivity as well as optimal sampling duration and sampling intervals. Lateral diffusion was assessed and blood samples were collected to look for potential systemic absorption. The dermal PK profile of a selected non-BE control product was also assessed to evaluate its suitability to serve as a negative control for BE relative to the reference product, Zovirax^®^ cream. dOFM samples were also used to inform about the calibration range of the analytical method (HPLC MS/MS) for acyclovir to be validated for its quantification in dOFM samples.

In the dOFM **verification main study**, 20 healthy participants were included at one clinical center (Medical University of Graz, Austria). Zovirax^®^ (Valeant, Bridgewater, NJ, United States) was used as the reference product (R) and Aciclovir^®^ (1A Pharma - Creme; 1A Pharma GmbH, Vienna, Austria) as the non-BE test product (T). R was compared to itself for BE assessment (R_1_ vs. R_2_) and R_1_ was also compared with T to show the discriminative ability of dOFM for non-BE products. Each participant had three application sites on each thigh with two dOFM probes inserted in each site. The application pattern of R (two sites, green/blue) and T (one site, red) was randomized on each thigh (Figure 1C). Dermal ISF was continuously sampled at 1 μL/min from a pre-dose baseline time period (-1 h to 0 h) until 36 h post-dose. dOFM samples with a volume of 20 µl were analyzed by using HPLC MS/MS to quantify acyclovir dISF levels. PK endpoints AUC and dermal C_max_ were log-transformed and assessed according to BE criteria where the 90% CI of the geometric mean ratio between the T and R has to fall within the limits of 80%–125% to confirm BE (US Food and Drug Administration, 2013).

**FIGURE 2:**
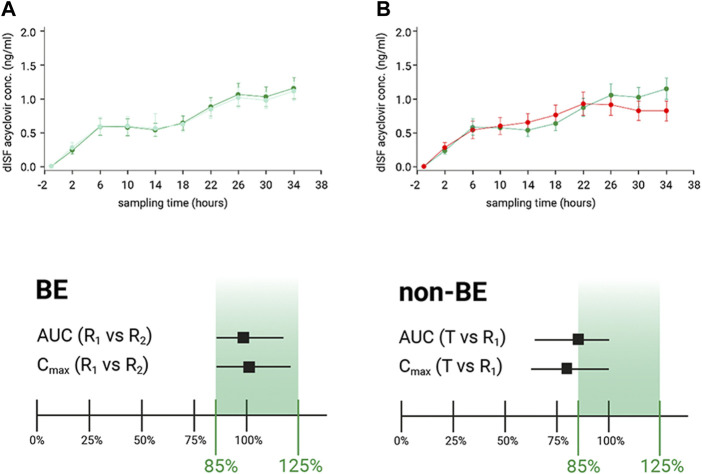
**(A)**: Dermal ISF concentration of acyclovir showed BE for the comparison of reference product (R_1_) to itself (R_2_). BE was confirmed as the calculated CI of the geometric mean ratios fell within the 80%–125% limits. **(B)**: Dermal ISF concentrations of acyclovir were different for the comparison of test product (T) with the reference product (R_1_), and non-BE was confirmed by the calculated CI of the geometric mean ratios that did not fall within the 80%–125% limits.

Results confirmed that dOFM was sensitive enough to confirm BE of R_1_ vs. R_2_ and to show that R_1_ vs. T did not fulfill the required BE criteria (Figure 2).

Even with a low number of participants and an API that has a low penetration rate and is present at very small concentrations in the skin, the main dOFM verification study successfully discriminated two different non-Q1 topical acyclovir cream products based on the conventional PK endpoints AUC and dermal C_max_. dOFM studies can thus be used to evaluate the relative bioavailability and BE of topical drug products (Bodenlenz et al., 2017).

## Summary and outlook

dOFM is an innovative PK-based method for BE assessment of topical drug products. dOFM has been evaluated as an alternative PK-based approach for BE assessment in two clinical BE verification studies up to now: one used acyclovir as a hydrophilic test drug (Bodenlenz et al., 2017) and the other one utilized a lidocaine/prilocaine combinational product with two moderately lipophilic topical drugs (Tiffner et al., 2020). dOFM has the potential to reduce or avoid clinical endpoint studies for topically applied drug products or any orally given drug products that act locally in the dermis. dOFM requires only 20 to 30 healthy participants for a clinical study with sufficient statistical power which drastically reduces the time and effort needed for the clinical study, and it provides an innovative alternative to avoid under-powered clinical endpoint studies (Zhu and Sun, 2019). Also, dOFM is not affected by placebo responses or by high variabilities due to difficulties in the objective assessment of treatment effects which are well-known limitations of clinical endpoint studies. Clinical PK-based dOFM studies offer an economical alternative to clinical endpoint studies and thus contribute to the development of affordable topically applied generic drug products. Since all dOFM setups have been developed in close cooperation with the respective regulatory agencies, dOFM is expected to become an integral part of international guidelines for the testing of dermal generic drug products.

Future applications include the use of dOFM in testing transdermal delivery systems that are designed to transport an API across the skin into deeper tissues and subsequently into systemic circulation. Transdermal drug products are topically applied but have their site of action elsewhere in the body. For testing of transdermal drug products, the dermal PK of the API can be assessed by dOFM. In combination with systemic PK in the blood, data about skin penetration and dermal clearance is obtained and can thus help to reduce or avoid clinical endpoint studies also for transdermal drug products. Furthermore, dOFM can be used to monitor biomarkers in the skin and provide information regarding skin irritation and skin sensitization which are required parameters in the development of transdermal drug products.
